# Bioceramics in Endodontics: Limitations and Future Innovations—A Review

**DOI:** 10.3390/dj13040157

**Published:** 2025-04-01

**Authors:** Peramune Arachchilage Amila Saman Prasad Kumara, Paul Roy Cooper, Peter Cathro, Maree Gould, George Dias, Jithendra Ratnayake

**Affiliations:** 1 Sir John Walsh Research Institute, Faculty of Dentistry, University of Otago, Dunedin 9016, New Zealand; amila.saman@postgrad.otago.ac.nz (P.A.A.S.P.K.); peter.cthro@otago.ac.nz (P.C.); maree.gould@otago.ac.nz (M.G.); 2 Department of Anatomy, School of Biomedical Sciences, University of Otago, Dunedin 9016, New Zealand; george.dias@otago.ac.nz

**Keywords:** endodontics, bioceramics, calcium silicate, calcium phosphate, bioactive glass, drawbacks

## Abstract

Bioceramic materials for endodontic treatments have gradually transformed over the years into materials with enhanced biocompatibility and chemical and mechanical properties compared to earlier generations. In endodontics procedures, these materials are used as restorative material in applications such as root-end fillings, pulp capping, perforations repair, and apexification repair procedures. However, they have far from ideal mechanical and handling properties, biocompatibility issues, aesthetic concerns due to tooth discolouration, limited antibacterial activity, and affordability, which are amongst several key limitations. Notably, bioceramic materials are popular due to their biocompatibility, sealing ability, and durability, consequently surpassing traditional materials such as gutta-percha and zinc oxide–eugenol sealers. A lack of recent advancements in the field, combined with nanomaterials, has improved the formulations of these materials to overcome these limitations. The existing literature emphasises the benefits of bioceramics while underreporting their poor mechanical properties, handling difficulties, cost, and various other drawbacks. The key gaps identified in the literature are the insufficient coverage of emerging materials, narrow scope, limited insights into future developments, and underreporting of failures and complications of the existing materials. Consequently, this review aims to highlight the key limitations of various endodontic materials, primarily focusing on calcium silicate, calcium phosphate, and bioactive glass-based materials, which are the most abundantly used materials in dentistry. Based on the literature, bioceramic materials in endodontics have significantly improved over recent years, with different combinations of materials and technology compared to earlier generations while preserving many of their original properties, with some having affordable costs. This review also identified key innovations that could shape the future of endodontic materials, highlighting the ongoing evolution and advancements in endodontic treatments.

## 1. Introduction

Bioceramic materials are biocompatible ceramic materials designed to interact with biological systems, which are primarily used in medicine and dentistry to replace or regenerate damaged hard tissues [1]. In endodontics, bioceramic materials primarily interact with dental pulp and the surrounding tooth and root canal system tissues, which are optimally designed for biocompatibility and mechanical properties [2]. With the introduction of mineral trioxide aggregate (MTA) as a bioceramic in 1993, bioceramics have transformed endodontics by providing solutions for repairing and reconstructing damaged dental tissues to restore their original functionality [3,4]. As of 2023, the global bioceramics market was valued at USD 7.4 billion. Within this market, the segment for root canal sealers is expected to grow at a compound annual growth rate (CAGR) of 3.6%, increasing from USD 2.41 billion in 2024 to USD 3.25 billion by the end of 2032 [5,6,7]. The market growth is primarily driven by endodontic applications, such as root canal fillings, sealers, and regenerative procedures in endodontics. With new innovations, materials such as calcium silicates, including MTA, bioactive glasses, and other calcium silicates, have shown the ability to promote the healing and regeneration of damaged tooth tissue [7].

**Figure 1:**
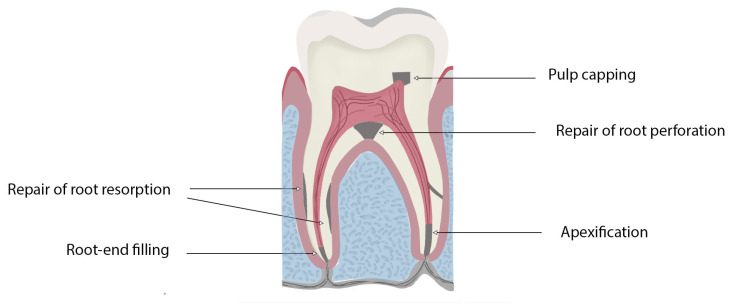
Schematic diagram highlighting the clinical applications of bioceramics for use in endodontics procedures.

Although bioceramics are among the most popular materials used in most countries, some regions still heavily rely on non-bioceramic options, such as zinc oxide–eugenol-based cements, as well as gutta-percha [8,9,10]. They are used for various endodontic applications as sealers, obturation materials, and materials for repairing perforations, as well as for pulp capping, pulpotomies, managing resorption, apexification, and regenerative treatments (Figure 1). Traditional endodontic materials, such as gutta-percha and zinc oxide–eugenol (ZOE) cements, exhibit significant limitations compared to modern bioceramic materials, particularly in terms of biocompatibility, bioactivity, and tissue regeneration potential. While gutta-percha serves as a standard obturation material, it lacks the ability to promote healing or actively integrate with surrounding tissues. Similarly, ZOE cements suffer from poor solubility, prolonged setting times, and dimensional shrinkage upon curing, which can compromise root canal treatment sealing and long-term success. In contrast, bioceramic materials offer superior biological and physical properties, addressing these deficiencies and aligning with the demands of contemporary endodontic practice [11].

In restorative dentistry, bioceramic materials are used as dentin substitutes for pulp capping, treating dentin hypersensitivity, and enabling dentin remineralisation [12,13]. Despite their many properties, bioceramics still exhibit some limitations.

Bioceramic materials in endodontics have been classified in many ways based on their applications, bioactivity, and the primary component of the material [2,14,15]. These materials are widely employed in combination, and zirconia and alumina are primarily used in endodontic materials as filler materials and radiopacifiers in various formulations [2,16].

Despite recent advancements in nanotechnology, bioceramics designed for endodontic applications are still far from ideal, highlighting the need for continued innovation to address their inherent limitations. While nanoparticles enhance the properties of endodontic materials, they also introduce additional costs and manufacturing complexities. Common endodontic materials use silver, silica, zinc oxide, iron oxide, nanopolymers, and carbon-based nanoparticles such as carbon nanotubes. However, these nanoparticles may pose potential toxicity risks and require thorough evaluation for cytotoxicity and biocompatibility. As such, long-term clinical data will be necessary to assess the safety and effectiveness of these nanomaterials [17].

**Figure 2:**
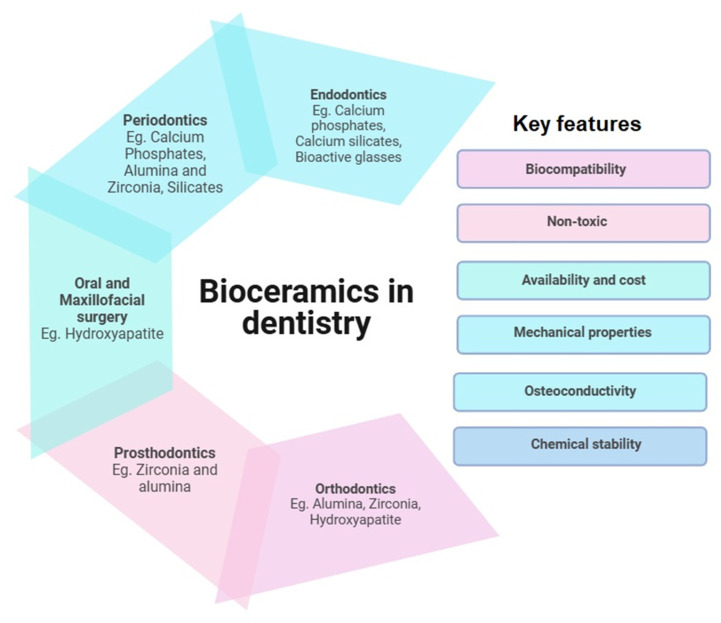
Applications of bioceramics in dentistry: key component and features.

While most literature aims at discussing the properties and comparative studies of bioceramic materials [18,19], the main objective of this review is to identify the limitations of current endodontic materials, explore their development and improvement challenges, and examine the strategies used to address these issues. The insights gained could guide future researchers and developers in advancing the endodontic material arsenal. Figure 2 depicts the applications of bioceramics in dentistry alone, with examples and key features suitable for their applications.

## 2. Bioceramic Materials

### 2.1. Calcium Silicates

Bioceramics used in endodontics are generally bioactive, amongst which calcium silicate-based cement (CSC) represents the most promising and frequently used due to its bioactivity, sealing ability, biocompatibility, and osteoinductivity [19,20,21]. In addition, calcium silicates also add value to endodontic materials, which can elevate pH and antibacterial properties [22]. CSCs can be classified based on their material chemistry and chemical composition. Among CSCs, tricalcium silicate and dicalcium silicate are the primary components; for example, mineral trioxide aggregate (MTA) combines tricalcium silicate and dicalcium silicates with other minerals. MTA is the first commercially available calcium silicate cement used in pulp capping. Its composition is almost identical to Portland cement, a material widely used in the construction industry, but adapted for endodontic therapy with refined materials and additives to improve its properties [23]. MTA is among the most widely used bioceramic in endodontics and is considered the gold standard due to its excellent biocompatibility, sealing ability, antimicrobial properties due to increased pH, and ability to promote healing peripheral tissues [21]. In addition, it has appropriate mechanical and bioactive properties that support its utility and make it one of the most versatile materials for use in endodontic procedures [24,25].

Endosequence (Brasseler, Savannah, GA, USA) and Biodentine (Septodont, Saint-Maur-des-Fossés, France) are two widely used endodontic materials, which also include tricalcium and dicalcium silicate. While these products have varying properties and applications, MTA remains the gold standard material for endodontics [26]. Table 1 highlights the most widely used calcium silicate materials for endodontic applications and their composition, applications, properties, and major drawbacks. Table 1 shows the calcium silicate-based bioceramics used in endodontic procedures.

### 2.2. Calcium Phosphates

Calcium phosphate-based dental materials are pivotal in modern restorative and regenerative dentistry due to their biocompatibility and similarity to the natural tooth mineral content [43]. Amongst these materials, hydroxyapatite, tricalcium phosphate, and dicalcium phosphate stand out for their unique properties and applications.

#### 2.2.1. Hydroxyapatite

Hydroxyapatite (HA) is the most abundant mineral in bone and teeth and is known for its excellent integration with natural bone, making it a popular choice for bone grafts, dental cements, and dental implants [44,45]. HA is a form of calcium phosphate that has the chemical formula Ca_10_(PO_4_)_6_(OH)_2_ and consequently consists of calcium (Ca^2+^), phosphate (PO_4_^3−^), and hydroxide (OH^−^) ions [46]. Similar to other calcium phosphate-based materials, HA is biocompatible and induces osseointegration and osteoconduction [47]. HA has been traditionally used as a coating for dental and orthopaedic implants, and modern endodontic applications include periapical defect repair, pulp capping, formation of apical barriers, and reparation of mechanical bifurcation perforations [47]. HA has also been used as a component of root canal-filling material in animal studies, where subsequent evidence of osteoconduction and stability were demonstrated [48]. Although HA shows greater biocompatibility, incorporating it into endodontic materials presents challenges, as it has been reported to weaken the material’s mechanical properties [49]. Studies have shown that HA reduces compressive strength crack initiation and increases porosity due to the solubility of hydroxyapatite-based materials and challenges in fine-tuning degradation, which could ultimately lead to microleakage. These issues represent some of the challenges in adapting this material for use in clinical applications [44,50,51].

#### 2.2.2. Dicalcium and Tricalcium Phosphates

With its varying solubility, tricalcium phosphate offers a controlled release of calcium and phosphate ions, enhancing bone regeneration and repair. Dicalcium phosphate, known for its rapid resorption rate, is often used in tooth-coloured filling materials and as a component in preventive dental treatment materials, aiding in remineralisation and preventing caries from forming [47]. Together, these calcium phosphate compounds provide a versatile toolkit for enhancing dental health and addressing a range of restorative needs.

Both tricalcium phosphate and dicalcium phosphate are equally valuable materials for use in bone regeneration and other dental applications. Tricalcium phosphate, in particular, acts as an osteoconductive material, promoting remineralisation and growth. This property has been utilised in dentistry for its use as a capping agent, apical barrier, and apexification treatments. It has also been tested in periodontal defect repair procedures. However, poor mechanical properties, low resistance to cracking, and unpredictable solubility may pose challenges when developing dental composite materials. Compared with Tricalcium phosphates, dicalcium phosphate dissolves relatively slowly and may be preferred where structural integrity is required [47].

In 1971, Hench developed a glass ceramic containing calcium and phosphate, known as bioglass, which demonstrated the formation of a chemical bond with host bone tissue through a calcium phosphate-rich layer [52]. Active restorative materials containing amorphous calcium phosphate (ACP) encapsulated in a polymer binder as a filler, were developed to promote tooth structure repair by sustaining significant amounts of calcium and phosphate ions [14]. In endodontics, calcium phosphate-based bioceramics are classified into calcium phosphate-based or mixtures of calcium silicate and phosphate [2]. Details of the most widely used calcium phosphate-based endodontic materials are provided in Table 2. 

### 2.3. Bioactive Glasses

Bioactive glass (BG) is primarily composed of silicon dioxide (SiO_2_), calcium oxide (CaO), sodium oxide (Na_2_O), phosphate (P_2_O_5_), and borate (B_2_O_3_), which are distinguished by their ability to release ions and form a hydroxyapatite layer at tissue interfaces [62,63,64,65]. BGs are often incorporated into materials to overcome the limitations of commercially available bioceramic sealers. Due to their non-crystalline structure, these particles are expected to elicit improved bioactivity compared with crystalline bioceramics, such as MTA and iRoot BP Plus, in procedures such as pulp capping [66]. Experimental studies have demonstrated strong antimicrobial properties due to the ability of BGs to raise the pH and calcium levels in the surrounding local tissue environment, enabling disinfection [67]. Notably, BGs can be classified into silicate-based glass (SiO_2_), borate-based glass (B_2_O3), and phosphate-based glass (P_2_O_5_) [67]. BGs initially form a silica layer allowing for calcium and phosphate ions to react, forming a calcium phosphate layer and ultimately forming HA over time and stimulating osteoblasts to proliferate and differentiate, enabling the synthesis and deposition of bone matrix [67,68]. The first and most studied BG is Bioglass 45S5, which is known for releasing relatively high quantities of phosphate, calcium, silicon, and sodium ions, promoting hard tissue formation [69]. Bioglass appears in various forms, including particulate, powder, mesh, cones, and pellets. It is often used in clinical applications such as grafting materials, endosseous implants, remineralisation, antibacterial agents, and as a medium for drug delivery. Bioglass is extensively applied in dentistry and orthopaedic fields and is a key component in dental materials, especially in endodontic composites [70,71].

Incorporating bioactive particles Niobophosphate (NbG) or BG 45S5 into endodontic cement shows promise in neutralising acidic environments and promoting hydroxyapatite precursor formation [69]. Clinically, this could create a bactericidal cement that facilitates tissue healing. Improved radiopacity and flowability would also aid in visualising the material on radiographs and filling complex root canal anatomies. However, the drawbacks include excessive weight loss and post-setting cytotoxicity, which may lead to cement degradation and tissue irritation [69]. The details for current bioactive glass-based endodontic bioceramic materials and their properties are summarised in Table 3. 

## 3. Limitations of Bioceramic Materials

### 3.1. Tooth Discolouration

**Figure 3:**
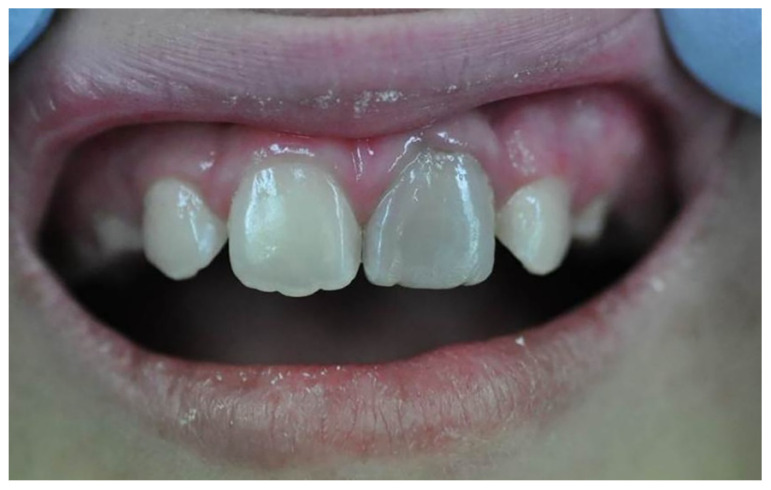
Clinical image of tooth discolouration attributed to the use of MTA [86].

Tooth discolouration (Figure 3) is a common aesthetic complication, especially occurring in anterior teeth due to pathological conditions and the materials used in root canal treatments [84]. Remnants of the pulp, micro-leakage, internal absorption, and treatment failure are some of the key contributing factors to tooth discolouration. Tooth discolouration can also occur following endodontic treatment when components of endodontic materials, such as bismuth, silver, and iron, react with the surrounding environment. With respect to bismuth oxide, a common radiopacifier included in calcium silicate materials such as MTA reacts with irrigation fluids such as sodium hypochlorite, blood, and dentin collagen to form brown or greyish precipitates. Upon exposure, bismuth oxide in the MTA rapidly reacts with NaOCl to form a dark brown-black sodium bismuthate (NaBiO_3_). Bismuth oxide has also been reported to react with blood, resulting in dark-coloured products. When bismuth oxide reacts with blood components, haemoglobin and hematin via redox reactions, the resulting products can infiltrate dentinal tubules and the crown [85].

The tooth discolouration associated with calcium silicate-based materials can start to appear as early as 3 months after placement, and the gradual increase has been documented for up to 2 years [87]. Among the widely used MTA types, Angelus and ProProot MTA were reported to impart severe discolouration that considerably negatively affects the aesthetics of the tooth [87]. NeoMTA, EndoSequence bioceramic putty, and Biodentine did not normally exhibit clinically perceptible colour changes due to the usage of either tantalum oxide or zirconium dioxide and thus might be considered for use in the aesthetic zone [27,34].

However, some studies indicated that alternative radiopacifiers, such as zirconium oxide and calcium tungstate, can also show colour alterations, albeit at lower levels than bismuth oxide containing MTA [88]. Bioroot RCS and TotalFill BC Sealer, MTA Bio-C Pulpo, MTA Vitalcem, Medcem MTA, TMR MTA, EndocemZr, and Rootdent have been formulated with zirconium oxide, while MTA HP and PD MTA White with calcium tungstate are purported to be non-staining [89]. However, tooth discolouration was investigated in a three-year in vitro study, and it was concluded that the discolouration effect was within acceptable limits [90]. This study further indicated that irrigation with saline or distilled water following NaOCl irrigation resulted in lower tooth discolouration levels [91]. The usage of a radiopacifer has been illustrated in Table 1.

### 3.2. Relatively Long Setting Time of Bioceramic Materials

Long setting times associated with conventional endodontic cements are a significant drawback in endodontic treatments, where rapid sealing and bonding are essential to prevent reinfection and minimise treatment time, thereby reducing patient discomfort and treatment costs. The ideal setting time for many clinical endodontic applications ranges from 3 to 10 min [92]. For instance, apical surgery demands the shortest setting time, possibly due to the risk of wash-out caused by blood flow. Novel calcium silicate products, such as Biodentine, MTA Plus, and light-curable TheraCal, offer relatively shorter setting times, addressing these challenges effectively.

Calcium silicate-based hydraulic cements, primarily composed of calcium trisilicate and calcium disilicate, undergo a hydration reaction with moisture from the surrounding environment and tissues [93]. The setting of calcium trisilicates is the main contributor to setting time in calcium silicate-based materials, whereas a much slower dicalcium silicate reaction provides secondary strengthening [92]. The setting reaction of these materials depends on the composition of the material, the type of chemical reaction involved, and differences in particle size, crystallinity, pH, and temperature [89,93]. Out of all the calcium silicate-based endodontic materials, MTA has a relatively slow setting time of approximately 3–4 h and takes 21 days for the complete curing process under an ambient temperature [94]. The setting time also differs between the different MTA formulations and depends on the composition and powder-to-water ratio.

As highlighted, particle size is one of the major contributors to the longer MTA setting time. Contemporary MTAs contain larger particle sizes, which results in the disadvantage of slow setting time [89]. There have been many studies that have attempted to overcome the long setting time, which is a notable disadvantage. Since MTA root fillings are considered permanent, the fully set material becomes highly dense, making its removal difficult and time-consuming. [94]. Therefore, achieving a balance between hardness, improved setting time, and biocompatibility needs to be carefully considered, and this depends on the particular application.

### 3.3. Handling Properties of Bioceramic Materials

While bioceramics offer significant benefits in endodontics, different materials exhibit a range of handling challenges before, during, and after application, many of which remain unresolved. For instance, calcium silicate-based materials like mineral trioxide aggregate (MTA) have historically presented difficulties due to the consistency of the set material. However, newer formulations—such as MTA Angelus, MTA Plus, NeoMTA, and Biodentine—have addressed these issues through innovative modifications. The incorporation of additives like polymers, plasticisers, and hydrosoluble gels into these formulations has significantly improved their handling properties [89].

Key issues include placement and workability, prolonged setting times, and, critically, difficulty in removal during retreatment or procedural revisions [94,95].

Removal challenges are particularly pronounced with calcium silicate-based bioceramics like MTA and newer bioceramic sealers.

### 3.4. Mechanical Properties of Bioceramic Materials

Calcium silicate-based MTA materials currently in use have shown adequate compressive strength values. After 24 h of setting, the compressive strength is generally between 40 Mpa and 60–70 Mpa after 21 days [96]. However, different formulations of MTA exhibit a range of values depending on the composition of these materials [97]. The values of similar formulations depending on the particle size have also shown variations. MTA angelus, a second-generation MTA formulation, has shown lower compressive strength values [98]. Even though MTA angelus has shown lower mechanical properties due to the exclusion of calcium sulphate (gypsum) in its formulation, it has shown an improved setting time of less than 50 min, whereas ProRoot MTA was reported to take 2 h, which is clinically less desirable [99].

Standalone calcium phosphates, including HA, are not as well explored or clinically adopted as calcium silicates for endodontic applications, primarily due to their poor mechanical properties, such as lower compressive strength and longer setting times. Though they are not yet mainstream, there have been reports of calcium phosphate-based endodontic materials developed for clinical use. Although these materials are not yet widely adopted in clinical practice, notable developments have been in this field. One example is the Chitra-CPC sealer, a locally developed bioceramic material created by the Sree Chitra Tirunal Institute for Medical Sciences and Technology (SCTIMST) in India. Initially designed for use as a bone graft and perforation repair material, the Chitra-CPC sealer has demonstrated potential in endodontics by reinforcing root strength and exhibiting excellent biocompatibility [100]. However, modified endodontic cements that include hydroxyapatite, calcium silicates, or Portland cement have shown variable material properties [28,50,101].

Bioactive glass is frequently added to different formulations or composites used in endodontics to provide various functionalities. Glass ionomers often benefit from adding bioactive glasses. Incorporating bioactive glass into glass ionomers has demonstrated that it can induce precipitates on the surface of demineralised dentin—however, the mechanical properties, such as compressive strength, vary along with the different formulations employed [102].

### 3.5. Shrinkage Properties of Bioceramic Materials

Shrinkage with respect to bioceramics is the decrease in volume when a bioceramic material undergoes a chemical reaction leading to its hardening. The polymerisation shrinkage is particularly significant in the setting reaction of an endodontic cement utilised in root canal procedures that include polymers or resins, making it a critical aspect to consider [103].

Calcium silicates undergo hydration reactions to form calcium–silicate–hydrate (C–S–H) gels and calcium hydroxide. The process is water-dependent, and incomplete hydration or moisture loss can result in volumetric shrinkage. Shrinkage is exacerbated under certain environmental conditions, such as acidic pH, which accelerates material dissolution and disrupts the hydration reaction [104,105]. Notable examples include silicate-based materials such as NeoPUTTY (NeoMTA 2) and MTA HP, which exhibited significant volumetric reductions in acidic environments, as shown via micro-computed tomography (micro-CT) [105,106].

With respect to calcium phosphates, their setting relies on dissolution–precipitation to form hydroxyapatite. Calcium phosphate materials can exhibit negligible shrinkage or slight expansion depending on ion concentrations and hydration conditions [92,107]. However, desiccation may cause porosity or small shrinkage-related defects under poorly controlled hydration, affecting dimensional stability [92].

Bioactive glass, on the other hand, exhibits minimal shrinkage (<1%) due to its robust dimensional stability in hydrated environments [92]. In contrast, resin-modified glass ionomers experience polymerisation during the acid–base setting reaction, causing significant volumetric shrinkage (0.5–6%) [92,108]. This shrinkage is associated with marginal gaps and secondary failures in some endodontic applications [92,108]. Table 4 summarises the volumetric changes or the shrinkage of different types of materials and their impact on clinical settings. 

### 3.6. Biocompatibility and Cytotoxicity of Bioceramic Materials

Calcium silicate cements, often used in endodontics and restorative applications, are generally well regarded for their biocompatibility and ability to promote tooth structure repair. However, some cytotoxicity can occur due to the formulation, setting conditions, and the release of residual chemicals. The biocompatibility and cytotoxicity of these materials, primarily MTA, Biodentine and BioRoot RCS (Septodent, Saint-Maur-des-Fossés, France) in clinically relevant settings are well established. Systematic reviews, such as those by Oliveira et al. [111] and Maru et al. [112], have consolidated available data, demonstrating consistently favourable biocompatibility profiles, particularly when compared to traditional materials such as resin-based sealers. The data emphasises both in vitro and in vivo investigations, which support calcium silicate-based materials’ ability to promote cell viability and minimise inflammatory responses.

Mineral trioxide aggregate (MTA) exhibits transient cytotoxicity and localised inflammation primarily during its initial setting phase, attributed to its high pH and release of components like calcium hydroxide and bismuth oxide, with occasional concerns about systemic toxicity observed in animal models; however, its long-term biocompatibility remains favourable [113,114,115].

On the other hand, the use of bioactive glass types, such as Bioglass^®^ 45S5 (NovaMin Technology, GlaxoSmithKline, Alachua, FL, USA), could lead to harmful effects on cells as a result of a significant increase in pH from the leakage of high levels of Na^+^ and Ca^2+^, which might also delay the formation of hydroxyapatite [116].

### 3.7. Microleakage

In endodontics, the passage of fluids, bacteria, or other substances in and out of the root canal system is called leakage. Microleakage is a known drawback of some endodontic materials. This process permits the entry of bacteria or fluids into the root canal system, leading to treatment failure and ongoing infection and inflammation. Leakage often occurs at a much smaller scale, which is often referred to as microleakage, and often at the interface between the endodontic material and the tooth structure. [117]. The movement of fluid, bacteria, and other substances, such as toxins, potentially results in long-term and short-term complications such as inflammation and reinfection. Modern endodontic treatments heavily rely on establishing an obturated root canal that is devoid of apical leakage.

Bioceramics that are used in endodontics undergo chemical degradation, particularly in a lower pH environment, are the main contributors to microleakage. Calcium silicate-based sealers undergo hydration-dependent setting reactions, which result in ion leaching over time under acidic pH conditions, and this leads to solubility complications affecting the mechanical integrity of the material [118]. However, the formation of HA on these materials may reduce this early stage degradation by inducing a mineral infiltration zone, promoting sealing [119].

In addition, the bond between endodontic sealers or root canal filling materials and the dentinal walls is crucial for proper sealing. This bond is essential for establishing a barrier against bacteria, thereby preventing reinfection. Studies have shown that resin-based sealers like AH Plus have better bonding strength than silicate-based bioceramic sealers [120].

While more stable in neutral conditions, calcium phosphate-based materials demonstrate increased solubility under acidic conditions and often show porosity or dissolution-related issues during the setting phase [118,121]. However, the mechanisms of degradation for calcium phosphate-based sealers remain comparatively understudied.

Endosequence BC Sealer is an endodontic material containing calcium silicates and calcium phosphates, and it has been shown to have more apical and coronal leakage than AH Plus, especially when used with the single cone technique (ref). Studies suggest that AH Plus forms a stronger bond due to its covalent interaction with dentin, resulting in less leakage.

Calcium phosphate monobasic present in the TotalFill^®^ BC Sealer™ (FKG Dentaire Sàrl, Le Crêt-du-Locle, Switzerland) has been shown to reduce calcium ion release. Indeed, TotalFill^®^ BC Sealer™ has a lower calcium ion release than BioRoot™ RCS. Other than the additives that limit the formation of the calcium hydroxide, the hydration of the premixed sealers depends on the water availability in the surroundings and the ion release through diffusion, which further delays or restricts the availability of calcium [108,122].

### 3.8. Solubility of Bioceramic Materials

The solubility of endodontic bioceramic materials can affect the materials’ overall clinical properties [123]. The solubility compromises the sealing ability, creating and increasing the space, potentially leading to treatment failure. It is recommended that a 3% maximum solubility be permitted in ISO 6876:2012 [124] root canal sealing materials to address this issue [123]. Notably, tricalcium phosphates have unpredictable fluid solubility [47]. BC-Endosequence (Brasseler USA, Savannah, GA, USA) bioceramic sealers, which have calcium phosphates as a component, have shown considerable (4.1%) solubility after 21 days when submerged in distilled water, and these values are much more favourable when compared with silicate-based endodontic materials such as ProRoot MTA [125].

### 3.9. Radiopacity of Bioceramic Materials

Radiopacity remains a critical concern in bioceramic-based endodontic materials due to variability in compliance with ISO 6876 and ADA Specification No. 57 standards [126]. A material is considered radiopaque if its ability to block X-rays matches or exceeds that of a 1 mm-thick aluminium (Al) sheet. The endodontic materials should exhibit minimum radiopacity requirements of 3 mm or 2 mm Al thickness, as the ISO and ADA standards specified. Insufficient radiopacity can lead to significant clinical challenges, including distinguishing materials from surrounding anatomic structures during radiographic evaluation, as observed with products like MTA Fillapex and Biodentine [1,2,5]. Several commonly used materials fail to meet these benchmarks under standard testing conditions. For example, MTA Fillapex often exhibits insufficient radiopacity due to its zirconium oxide-based formulation, which provides lower radiopacity than alternatives such as bismuth oxide [127,128,129]. Similarly, Biodentine has also shown mixed results; though compliant in some studies, it has failed to meet standards in others, with radiopacity as low as 2.8 mm Al under traditional test conditions, highlighting its borderline adequacy [130,131]. EndoSequence BC Sealer and Bio-C Sealer demonstrate radiopacity values slightly above the 3 mm Al ISO threshold but remain notably less radiopaque than epoxy-based sealers such as AH Plus, raising concerns regarding the clinical suitability under certain situations [128,132]. MTA Angelus (white/grey) meets or minimally exceeds ISO standards but demonstrates variability based on preparation techniques and testing methods, such as differences in powder–liquid ratios [129,133,134]. Conversely, formulations such as TotalFill BC Sealer HiFlow consistently achieve robust radiopacity beyond ISO benchmarks, attributed to the effective incorporation of bismuth oxide in optimal proportions [135,136,137]. Radiopacity inconsistencies are associated with factors such as radiopacifier type and concentration, variability in material dispersion, and testing protocol differences. For example, testing under real-world conditions, such as tissue-simulator models, has demonstrated that materials such as Biodentine may perform better than in standardised tests, suggesting limitations in the current ISO/ADA analysis methods [131]. However, the continued use of materials with borderline radiopacity values and limitations in current radiopacity standards underscores the need for improved formulations and more clinically relevant testing methods.

### 3.10. Antibacterial Properties of Bioceramic Materials

Calcium silicate-based endodontic materials’ antibacterial properties are mainly attributed to their increased pH, calcium ion release, and calcium hydroxide formation [138]. However, unlike calcium silicate (CS)-based materials, calcium phosphate cements do not impart antibacterial properties [139]. Providing antibacterial properties in most endodontic treatments would be recommended to eliminate bacteria from the root canal system, prevent reinfection, and offer a favourable healing environment. Antibacterial properties are particularly critical in combating persistent endodontic pathogens such as *Enterococcus faecalis*, *Porphyromonas gingivalis*, and *Fusobacterium nucleatum*, which are implicated in secondary infections and treatment failures [140,141]

Among endodontic materials, calcium silicate-based materials such as MTA are reported to have significant antibacterial properties. However, higher activity has been reported, particularly when they are freshly prepared and mixed. [141,142,143,144]. The change in activity has been attributed to the decrease in the pH of the media over time [145,146]. In addition, the activity has been reported to be dependent on the agar disk diffusion method employed. In contrast, direct methods have shown higher activity due to the easy diffusion of the ions [147].

Endodontic materials based on calcium silicates, such as MTA, Biodentine, NeoSealer Flo, and BioRoot RCS, have shown varying levels of antibacterial activity against different bacteria. Among these materials, Biodentine has consistently shown strong activity against Enterococcus faecalis and Streptococcus mutans, which is attributed to its ability to create a higher pH in the medium [148,149]. However, studies also suggest that the activity diminishes over time against biofilms. On the other hand, MTA has shown moderate activity against Enterococcus faecalis and Streptococcus mutans compared to Biodentine and newer endodontic sealers [150]. The literature suggests that silicate-based materials are effective against planktonic E. faecalis and S. mutans but show limited candida-causing Candida albicans [148]. Based on recent studies, emerging materials with modified nanoparticles, antimicrobial materials such as chitosan, or substitutions with antimicrobial elements show greater efficacy against managing bacterial strains that are resistant to conventional materials.

### 3.11. Affordability and Cost

Numerous bioceramic materials under different names are used for endodontic applications that incur varying costs. However, a direct comparative study of the cost of the materials or treatment has not been properly conducted in the recent past. The limited data (Table 5) suggest that Biodentine is more affordable and has a faster setting time; easiness of manipulation has become a popular choice among dentists. TheraCal LC, a resin-modified calcium silicate, also has a lower cost. However, a detailed analysis of effectiveness over cost requires in-depth analysis and more data for a proper analysis [151].

## 4. Future Advances in Bioceramics for Use in Endodontics

Future advances in bioceramics for use in endodontic treatment should involve enhancing the material’s properties, exploring combined applications, and tailoring treatments. Indeed, research needs to concentrate on refining these materials’ physical, chemical, and biological characteristics [152].

In the advancement of bioceramic materials, nanotechnology plays a pivotal role. The incorporation of nanomaterials into endodontic materials can enhance properties such as biocompatibility, bioactivity, mechanical strength, radiopacity, sealing ability, ease of handling, setting time, and, notably, antibacterial effects.

The mechanical properties of endodontic materials have been tested and enhanced through the incorporation of various nanomaterials. Carbon nanotubes; nanoclays such as montmorillonite, nano-calcium hydroxide, zinc oxide, aluminate, and silicon dioxide; and numerous other nanopowders have demonstrated improved mechanical properties when used as reinforcements [153,154,155]. In addition, nanoparticles have been shown to reduce leakage and improve the sealability of endodontic materials [156]. Antibacterial property is an important property of the endodontic materials for the prevention of reinfection of the treated tooth. Many nanoparticles, such as silver, zinc oxide, zirconium oxide, and titanium oxide, as well as many forms of nanomaterials, have been tested and incorporated to increase the antibacterial properties of endodontic materials [157,158]. Furthermore, some nanomaterials, such as montmorillonite have been tested for their ability to deliver drug molecules to specific targets, potentially increasing the efficacy of drugs designed for smart, targeted delivery [154]. Nano-hydroxyapatite, an important material and a primary component of dental structures, has been utilised in various ways to enhance the biocompatibility and osteogenicity of endodontic materials [159].

Employed nanomaterials have been incorporated as additives to improve and impart various properties. Materials such as silver oxide, zinc oxide, and many others can be synthesised separately and incorporated into endodontic materials. Some materials are incorporated using chemical methods [160], while others are incorporated through intercalation and doping [161], such as on the surface of nanoclays [154]. Additionally, these materials can be incorporated into polymer-based materials to enhance and impart various properties.

In addition to nanomaterials, numerous innovations have been explored to enhance the properties of endodontic materials. Several approaches that have been tested, implemented, or that show potential for improvement are outlined below.

### 4.1. Radiopacifiers

Tooth discolouration associated with endodontic material has been one of the major issues over the years. Calcium silicate-based MTA and related materials often included bismuth oxide as a radiopacifier, resulting in black or brown precipitation upon interaction with the surrounding environment. With the introduction of newer materials, tooth discolouration has been addressed with alternative radiopacifers such as zirconium oxide, zinc oxide, and tantalum oxide. Zirconium oxide-containing materials, such as EndocemZr [ECZ], are now available for clinical use, with low tooth discolouration levels; however, longer-term studies may be required to confirm this [162]. In addition, novel radiopacifiers have been studied that exhibit decreased tooth discolouration effects. Among these materials, MTA-like cement, created by incorporating 30% barium titanate and set with a 10% CaCl2 solution, demonstrated a radiopacity of 3.68 ± 0.24 mm Al, a diametral tensile strength (DTS) of 2.54 ± 0.28 MPa, and initial and final setting times of 55 and 23 min, respectively. Notably, the radiopacity was marginally increased when compared with the setting using deionised water. Additionally, this innovative MTA showed outstanding colour stability and excellent biocompatibility, making it suitable for future commercial use in endodontic treatments [83].

### 4.2. Handling Properties

In recent years, sol–gel methods for developing endodontic cement have gained attention with precise control over the material’s composition, microstructure, and texture, making it a highly promising avenue for advanced endodontic material in the upcoming years [163].

At the same time, various modifications have been introduced for calcium silicate-based materials, such as Generex A (Dentsply Tulsa Dental Specialties, Tulsa, OK, USA), which is one such formulation developed some time ago and is a calcium-silicate-based material similar to ProRoot MTA but mixed with gels instead of water. Generex A shows a dough-like consistency, making it easier to shape into a rope-like mass, which improves its handling properties. Generex A is also reported to have minimal tissue sensitivity, promote cell proliferation beside MTA, and facilitate nodule formation [164].

### 4.3. Biocompatibility

When discussing new developments in endodontic materials, HA has recently gained prominence in the development of bioceramic materials for endodontic applications. HA has excellent biocompatibility, bioactivity, osteoconductivity, and physical and chemical properties, making it well placed for use in dental bioceramics [165]. Recent studies have shown that natural HA derived from animal tissue, such as from bovine bone, has shown higher biocompatibility and is a viable, cheaper alternative to synthetic hydroxyapatite, which could easily be incorporated into dental bioceramics with lower associated costs and improved biocompatibility [166,167]. Bovine HA has already been tested in an experimental endodontic bioceramic material combined with ZrO_2_, and this Portland cement-based material demonstrated lower cytotoxicity and lower solubility, confirming that bovine or animal-derived HA has greater biocompatibility than synthetic hydroxyapatite-based endodontic cement [101]. As a waste product in the meat industry, this will further reduce the burden and environmental impact while providing cheaper and greener alternatives [166,167].

### 4.4. Mechanical Properties

Bioactive glass in dentistry can be incorporated into hydraulic ceramic and hydrophobic materials, making them clinically suitable novel materials with enhanced properties such as improved mechanical strength, sealing ability, remineralisation, and antibacterial activity [168]. Table 6 summarises the recent developments in endodontic bioceramics and addresses several limitations.

Studies investigating hydroxyapatite-modified endodontic cements have reported mixed outcomes regarding their mechanical properties, highlighting both potential benefits and trade-offs. Incorporating hydroxyapatite into calcium silicate-based cements, such as MTA or Portland cement, generally exhibit improved properties, such as increased compressive strength over extended curing periods [101], as well as a reduced setting time when Zn-doping of hydroxyapatite was used [28]. However, other modifications, such as nano-hydroxyapatite (10–20%), have seen reduced compressive strength [50] and have also resulted in increased porosity and solubility, suggesting that higher hydroxyapatite concentrations can negatively impact the mechanical properties. While hydroxyapatite-modified formulations demonstrated enhanced bioactivity and biocompatibility (e.g., improved cell viability and antibacterial activity) [101], these mechanical property compromises raise concerns regarding their long-term structural reliability. Importantly, studies in this area have focused mainly on compressive strength, with mechanical properties such as flexural strength and fracture toughness, while cyclic loading resistance remains underexplored [28,101]. While hydroxyapatite modification offers promising improvements in biological properties, further optimisation is required to address the current mechanical limitations and ensure their clinical applicability in endodontic treatments.

Calcium phosphate-based endodontic materials, such as tricalcium phosphate (TCP) or dicalcium phosphate (DCP), primarily aim to improve biocompatibility and ion release to support tissue healing and bioactivity. However, their mechanical properties, such as compressive strength, flexural strength, and fracture toughness, are consistently inferior to calcium silicate-based materials such as MTA or Biodentine. While doping with ions (e.g., strontium, copper, zinc) or combining with additives (e.g., PLA microspheres) can improve mechanical performance to some extent [1,3], these materials remain unsuitable for high-load or structurally demanding endodontic applications. Commercial examples like TheraCal LC and Calciblast highlight limited utility for specific cases such as in pulp capping or low-load sealing. Calcium silicate-based products with integrated calcium phosphate additives (e.g., Biodentine) often outperform purely calcium phosphate-based materials in mechanical and clinical metrics.

Bioactive glass is naturally prone to brittleness due to its glassy structure, which lacks the fracture toughness of certain ceramic materials. As a result, it is more vulnerable to fracturing when subjected to stress [169]. Future development should focus on hybrid materials or innovative composites that bridge the mechanical durability and bioactivity gap.

The broader implications of advancing endodontic materials with innovations like nanomaterials and by other means extend far beyond clinical efficacy, influencing cost, time efficiency, and accessibility in meaningful ways. While the initial development and integration of these materials may involve higher costs due to research and production, they promise long-term savings by reducing retreatment rates, minimising complications such as reinfections, and lowering the overall burden for patients and providers. From a clinical perspective, endodontic material advancement significantly enhances physicians’ procedural efficiency by providing optimal handling characteristics, rapid setting times, improved consistency, excellent radiopacity, and superior mechanical strength. These properties allow for precise application and adaptation within the complex root canal system, reducing chair time and increasing patient throughput in a busy practice. Moreover, their enhanced sealing capabilities and antimicrobial effects, which are often driven by nanomaterials, effectively eliminate residual bacteria and prevent microleakage, which are critical factors in minimising postoperative complications such as persistent infections or periapical pathology. This clinical reliability reduces the need for follow-up appointments, ensuring better treatment outcomes, streamlining patient management, and bolstering the overall success of endodontic therapy.

Patients benefit from accelerated healing, driven by bioactive nanomaterials that promote tissue regeneration, allowing for quicker recovery and fewer disruptions to daily life. Although adoption may initially be limited to specialists due to cost and training, as manufacturing scales and prices drop, these innovations could elevate the standard of care globally, particularly in underserved areas, balancing upfront investment with substantial long-term gains in efficiency, durability, and patient outcomes.

**Table 6:** Summary of recent developments of endodontic bioceramic materials with the potential to advance endodontic treatment outcomes.

Study	Limitations Addressed	Key Innovations/Modifications	Methods for Characterisation	Outcome	Reference
MTA, calcium silicates	Tooth discolouration, long setting time	Introduction of barium titanate (BTO) and calcium chloride	Radiopacity using X-ray device and setting time with Vicat needle	Increased radiopacity and shorter setting time	[83]
MTA, calcium silicates	Tooth discolouration	Addition of 5–45% zinc oxide	VITA Easyshade V digital spectrophotometer	Reduction in tooth discolouration without affecting other properties	[170]
MTA, calcium silicates, amoxicillin-loaded microspheres	Discolouration, long setting times, handling, antimicrobial properties	Amoxicillin loading, alternative radiopacifiers, improvements in handling tools	Handling evaluations, discolouration studies (alternative radiopacifiers), antimicrobial testing	Reduced discolouration; moderate antimicrobial effects; enhanced handling	[94]
MTA, calcium silicates, nanomaterials, hybrid calcium silicate–bioglass	Setting time, antimicrobial properties, brittleness	Nanoparticles (ZnO, TiO_2_, Ag), hybrid bioactive materials	Antibacterial testing, mechanical testing (brittleness), ion release, HA formation	Improved bioactivity and antimicrobial effects; moderate handling improvements	[20]
Generex A	Handling properties, improved osteogenic potential	MTA, mixed with gel material to create dough-like consistency that is easier to handle	NA	Improved handling properties	[2,164,171]
Sol–gel-derived MTA, bioceramics with ethanol post-treatment	Setting time, handling, bioactivity enhancement	Sol–gel synthesis, ethanol post-treatment for smaller particle size	Hydroxyapatite formation in SBF, SEM for surface analysis	Enhanced bioactivity (HA), reduced setting time, improved cohesion	[163]
Nanoparticle-modified MTA and bioglass	Long setting time, brittleness, antimicrobial properties	Zinc oxide (ZnO) particles, silver nanoparticles (AgNPs)	Antibacterial activity (disc diffusion), compression testing	Improved antimicrobial properties; brittleness reduced moderately; mechanical strength slightly improved	[172,173]
MTA, TotalFill, Biodentine	Discolouration, handling, setting times	Pre-mixed formulations, zirconium oxide as radiopacifier	Handling studies, discolouration observation	Reduced discolouration (zirconium oxide); faster setting times; enhanced usability	[174]
Sol–gel calcium silicate cements	Handling, setting times, bioactivity	Sol–gel method with post-synthesis ethanol treatment	Particle size analysis (XRD), bioactivity (HA formation), handling time comparisons	Finer particle size; faster setting times; enhanced bioactivity	[175]
MTA, Biodentine, Endobinder, Generex A	Setting time, biocompatibility	Modern alternatives to MTA; improved calcium silicate-based materials	Setting time evaluations, biocompatibility tests	Faster setting time; biocompatible formulations	[2,13]
Biodentine, TheraCal LC	Setting times, antibacterial properties, pulp capping	Light-curable formulations, pre-mixed formulations for easier handling	Antibacterial testing, cytotoxicity testing, setting-time measurement	Significantly faster setting time; reduced cytotoxicity	[175]
Calcium silicates, NeoMTA, Bio MTA+	Setting time, discolouration	Nano-hydroxyapatite reinforcement, alternate radiopacifiers	Cytocompatibility tests, SEM imaging, HA formation	Improved bioactivity and cytocompatibility	[176]
Bioceramic sealers with silver nanoparticles	Antimicrobial properties, sealing capacity	AgNPs for bacterial inhibition	Push-out bond strength tests, antimicrobial assays	Enhanced antimicrobial properties; limited improvement in bond strength	[177]

## 5. Conclusions

Bioceramics play a significant role in endodontic treatments, providing a biocompatible and bioactive solution that addresses many of the challenges of endodontic procedures. However, despite their beneficial biocompatibility and mechanical properties, none of the current products are ideal, and each has limitations. The literature has identified several key drawbacks, namely tooth discolouration, slower setting time, handling difficulties, and compatibility issues. Further work is therefore required in this field to develop improved bioceramic materials and address their current clinical limitations. As a narrative review, one of the main limitations identified is the overgeneralisation of the literature. Additionally, the existing studies have primarily focused on laboratory or in vitro findings, which may not fully translate to real-world clinical scenarios. In conclusion, while endodontic bioceramics have made significant progress in addressing specific drawbacks, the literature suggests that there is still room for improvement to increase their clinical utility.

## Figures and Tables

**Table 1:** Calcium silicate-based bioceramics used in endodontic procedures.

Brand Name	Core Ingredients	Radiopacifiers	Additives	Clinical Applications	Properties Evaluated	Drawbacks	Notable Observations	Reference
ProRoot MTA	Tricalcium silicate, dicalcium silicate, tricalcium aluminate, calcium sulphate	Bismuth oxide	N/A	Pulp capping, apexification, root-end filling, perforation repair	High bioactivity, sealing ability, antimicrobial activity with high pH	Discolouration from bismuth oxide, long setting time (78 min), handling difficulties	Gold standard material for endodontic procedures with excellent biocompatibility and antibacterial activity	[26]
Biodentine	Tricalcium silicate, dicalcium silicate, calcium oxide, calcium carbonate	Zirconium oxide	Calcium chloride (accelerator)	Pulp capping, apexification, perforation repair, dentin replacement	Improved aesthetics, shorter setting time, dentin-like mechanical properties, high bioactivity (apatite deposition)	Slightly lower radiopacity compared to bismuth oxide materials	Faster hydroxyapatite (HA) formation at the material–dentin interface avoids discolouration due to zirconium oxide	[27,28,29]
EndoSequence BC Sealer	Tricalcium silicate, dicalcium silicate	Zirconium oxide, tantalum oxide	N/A	Root canal fillings/sealing, pulp capping, apexification	Premixed versions improve handling and prevent contamination; high bioactivity	Potential long-term instability due to porosity	Tantalum oxide provides radiopacity while avoiding discolouration	[27,30,31,32]
BioRoot RCS	Tricalcium silicate, dicalcium silicate	Zirconium oxide	Calcium chloride	Root canal sealer, pulp capping	High solubility and prolonged alkalinity, affects long-term sealing ability	None specifically identified in the papers	High biocompatibility and excellent long-term sealing attributed to low solubility and stable formulations, mineralisation potential	[30,32,33,34]
iRoot BP/SP	Tricalcium silicate, dicalcium silicate	Zirconium oxide	N/A	Perforation repair, sealing, root-end fillings	High bioactivity, sealing ability	Lack of long-term clinical testing	Relies heavily on zirconium oxide for radiopacity and bioactivity	[27,33]
MTA Angelus	Tricalcium silicate, dicalcium silicate, tricalcium aluminate	Bismuth oxide	N/A	Pulp capping, dentin repair, apexification	Similar to ProRoot MTA but with a faster setting due to the lack of calcium sulphate, high calcium release, high porosity, and solubility	Discolouration due to bismuth oxide	Popular in Latin America; similar composition and applications to ProRoot MTA; high calcium release, high porosity, and solubility	[33,35,36]
Neo MTA Plus	Tricalcium silicate, dicalcium silicate, tricalcium aluminate, calcium sulphate	Tantalum oxide		Pulp capping, sealing, root-end filling perforation repair, apexification	Improved handling and reduced setting time due to added calcium chloride	Lack of long-term results	Tantalum oxide-based formulation avoids discolouration while improving clinical handling workflow. Biocompatibility similar to that of Biodentine and ProRoot MTA	[27,34,37]
Ceraseal sealer	Tricalcium silicate, dicalcium silicate	Zirconium oxide	Thickening agent	Root canal sealing	High bioactivity and sealing ability	Potential porosity affecting sealing over time	Showed good cell differentiation, mineralisation, and anti-inflammatory potential; also shows good sealing, but porosity may raise questions regarding long-term stability	[31,38]
MTA Fillapex	Calcium trisilicate, calcium disilicate, salicylate resin, salicylate resin, natural resin, silica	Bismuth oxide	Resin (to improve flow)	Root canal fillings/sealing	Easy handling and flow, lower bioactivity due to resin components	Reduced bioactivity and long-term issues with high solubility	Resin addition improves handling but sacrifices hydroxyapatite formation and long-term sealing potential	[27,34,39]
MM-MTA (MicroMega)	Tricalcium silicate, dicalcium silicate, tricalcium aluminate, bismuth oxide, calcium sulphate dehydrate, and magnesium oxide	Bismuth oxide	N/A	Root repair, pulp capping	Bioactivity and HA formation tested	Lack of structured clinical use documentation	Bioactive by forming apatite crystals; lower calcium release compared to ProRootMTA and Biodentine	[40]
BioAggregate	Tricalcium silicate, tantalum oxide, calcium phosphate, silicon dioxide	Tantalum oxide	N/A	Root canal sealing, apexification	High calcium ion release in the early stages, bioactive with dentin	Radiopacity is slightly lower compared to bismuth oxide materials	The absence of bismuth oxide prevents discolouration, making it more aesthetically friendly	[41]
TheraCal LC	Calcium trisilicate, calcium disilicate, Bis-GMA (Bisphenol A diglycidyl methacrylate), PEGDMA	Barium zirconate	Light-curing resin	Pulp capping, liner for crown, bridge placement	Good handling due to light-curing, reduced bioactivity compared to non-resin bioceramics	Low bonding strength to dentin	The resin significantly simplifies clinical application but limits bioactivity and calcium ion release	[35,42]

**Table 2:** Details and properties of calcium phosphate-based bioceramic materials used in endodontics.

Brand Name/ Product	Core Ingredients	Radiopacifiers	Additives	Clinical Applications	Properties Evaluated	Drawbacks	Notable Observations	Reference
CAPSEAL I/II	Tetracalciumphosphate Dicalcium phosphate, portland cement	Zirconium oxide	Sodium phosphate buffer	Root canal sealing, hard tissue deposition	Biocompatibility low cytotoxicity, Higher mineralisation, inflammatory response, sealing ability	Possible inflammatory response.	Good hard tissue deposition: inflammatory markers elevated compared to competitors (ARS, PCS).	[53,54]
Apatite Root Sealer (ARS I/III)	Tricalcium phosphate, Hydroxyapatite (HA), olyacrylic acid	Bismuth oxide	None specified	Root canal sealing	Inflammatory responses, sealing ability	Mechanical strength limitations	Outperformed zinc oxide–eugenol sealer in sealing ability; comparable to CAPSEAL, lower inflammatory response.	[53,55]
Smartpaste Bio	Hydroxyapatite, monobasic calcium phosphate, polymer base	Not specified	None specified	Obturation material	Hard tissue deposition, calcium ion release, mineralisation, antibacterial activity	Handling challenges	Supports mineral deposition; superior mineralisation compared to Acroseal and Sealapex and shows biocompatibility	[56]
Fully Injectable CPC (FI-CPC)	Calcium phosphate	Not specified	None Specified	Tooth perforation repair	Injectability, setting time, dimensional stability, non-toxic, non-allergic, non-pyrogenic, and soft-tissue compatible	Low compressive strength	High injectability through fine needles. Dimensional stability was achieved, but lacks clinical data on biocompatibility compared to marketed sealers.	[57]
Methacrylate-based Sealers	Nanostructured hydroxyapatite (HAp), α-TCP	Not specified	None specified	Root canal sealing, bioactive endodontics	Biocompatibility (MTT, SRB assays), bioactivity (ALP, Alizarin Red staining), antibacterial activity	α-TCP associated with lower cytocompatibility	HA-based compositions perform better in bioactivity compared to α-TCP. Lower compatibility with α-TCP due to cytotoxic concerns.	[58]
Glycerol Salicylate Sealer	Glycerol salicylate resin, Calcium hydroxide, α-TCP	Not specified	None specified	Root canal sealing	Mineral deposition, solubility, pH stability	Long setting time	Forms stable calcium phosphate layers: solubility reduced with increased α-TCP content but handling needs improvement due to prolonged setting.	[59]
Fluorapatite CPC	Calcium phosphate, sodium fluoride, tricalcium silicate	Not specified	Fluoride	Root canal repair, bioactivity enhancement	Bioactivity (hard tissue formation), sealing ability, setting time	Limited preclinical dental models	Fluorapatite enhances bioactivity and stability in acidic conditions but lacks head-to-head performance data compared to commercial MTA or Biodentine formulations.	[60]
CS/HA/β-TCP Composite	Hydroxyapatite, β-TCP	Not specified	Chitosan	Regenerative endodontic applications	Collagen formation, biodegradability, tissue regeneration (in vivo)	No clinical or handling data available	Promising tissue formation with chitosan integration for collagen type III; lacks sealing and degradation rate data when applied in endodontic contexts.	[61]

**Table 3:** Details of current bioactive glass containing bioceramics used in endodontics procedures.

Brand Name	Core Ingredients	Radiopacifier	Additives	Clinical Applications	Properties Evaluated	Drawbacks	Notable Observations	References
GuttaFlow Bioseal	Gutta-percha Polydimethylsiloxane (PDMS), platinum catalyserbioactive glass (CaO, SiO_2_, Na_2_O, P_2_O_5_)	Zirconium dioxide	None reported	Root canal sealing, biomineralisation	Dissolution, mineralisation, apatite formation, flowability, solubility, ion release	Low filler content, limited long-term data, alkaline pH; potential cytotoxicity under prolonged exposure	Exhibits hydroxyapatite formation, bacteriostatic effects; interacts well with gutta-percha cones	[62,72,73,74]
Nishika Canal Sealer BG	Fatty acid, calcium silicate glass, SiO_2_, MgO	Bismuth subcarbonate,	None reported	Root canal sealing, dentin bonding	Adhesion to root dentin, sealability, interfacial adaptation	Difficult removability, high pH causes some tissue irritation, setting dependent on moisture	Prominent cytocompatibility, osteogenecity, and angiogenecity	[62,75,76,77]
BioRoot RCS	Tricalcium silicate, zirconium oxide, calcium chloride, BG, povidone	Zirconium oxide	None reported	Root canal sealing, mineralisation, apexification	Ion release, radiopacity, sealing ability, antibacterial properties	High solubility compromising longevity	Prominent sealing ability with bioactive properties but prone to degradation in moist environments; more biocompatible than MTA	[75,78,79]
Biodentine	Tricalcium silicate, dicalcium silicate, calcium carbonate, BG-modified mix, iron oxide, hydro soluble-polymer	Zirconium oxide	Calcium chloride, fluoride, strontium (in modified versions)	Pulp capping, dentin remineralisation, apexification, root-end filling	Compressive strength, setting time, bond strength remineralisation	Solubility, high alkalinity, radiopacity issues in modified formulations, reduced mechanical properties, initial low cell viability	High remineralisation and bioactivity, promoting dentin regeneration and sealing, superior to MTA in biocompatibility and mineralisation	[80,81,82]
Bright Endo MTA Sealer	Calcium silicate (50–70%), methyl cellulose, BG	Bismuth oxide	BG nanoparticles (85% SiO_2_, 15% CaO)	Root canal sealing, osseous regeneration	Flowability, biocompatibility, antibacterial activity	Limited commercial use; insufficient data on long-term clinical outcomes	Nanoparticle BG improves flowability and antibacterial activity; bioactive ion release studied	[83]
Custom Nano 58S BG Sealer	Nano-tricalcium silicate, 58S BG (SiO_2_ 58%, CaO 33%, P_2_O_5_ 9%)	Zirconium dioxide	None reported	Root canal sealing	Interfacial adaptation, sealing ability, bioactivity	Experimental; lacks long-term clinical validation	Demonstrates improved interfacial bonding and adaptability compared to commercial sealers	[75]

**Table 4:** Shrinkage properties of different bioceramic materials.

Material Type	Mechanism of Dimensional Change	Relative Shrinkage	Influencing Factors	Clinical Impacts	References
Calcium silicates	Shrinkage due to hydration and water loss - Minor shrinkage (<2%)	Moderate (0.5–2%) in clinical environments	pH, hydration consistency, environmental moisture	- Marginal gaps due to shrinkage can lead to microleakage and bacterial ingress - No explicit clinical studies showing longitudinal failure related to shrinkage, but in vitro bacterial leakage studies confirm gap-associated risks	[104,105,109,110]
Calcium phosphates	Slight shrinkage or expansion due to crystallisation (precipitation)	Low (often negligible or slight expansion)	Hydration, ion concentration, supersaturation	- Rarely fail due to shrinkage when hydration is controlled - Potential risks of cracks or marginal defects due to desiccation-related porosity formation - Limited clinical studies directly testing shrinkage-related outcomes; primarily evaluated in in vitro models	[92,107]
Bioactive glass	Minimal shrinkage, primarily dimensionally stable	Low (<1%)	Dehydration, improper ratio of liquid–powder	- Highly dimensionally stable - Not directly correlated to clinical failures related to shrinkage - No significant clinical studies focused on volumetric behaviour in endodontics; research mainly limited to lab-based evaluations	[92]
Glass ionomers	Polymerisation shrinkage in resin-modified types Acid–base reactions in conventional forms produce minimal shrinkage	High (0.5–6%) for resin-modified formulations	Resin content, cavity thickness, curing stress	- Polymerisation shrinkage causes gaps that may contribute to microleakage - Clinical studies confirm that resin-modified glass ionomers have higher gap formation in endodontics compared to bioceramic materials	[92]

**Table 5:** Cost and estimated treatment cost for most popular endodontic materials.

Material Type	Material Unit Price (Approximate) USD	Cost per Treatment (Estimated)	Reference
ProRoot MTA	47 per 0.5 g	23.50 (High cost)	[151]
MTAAngelus	52 per 1 g	13 (Medium cost)	[151]
RetroMTA	14.50 per 0.3 g	14.50 (Medium cost)	[151]
Biodentine	10.50 per 0.7 g	10.50 (Medium cost)	[151]
TheraCalLC	21 per 1 g syringe	5 (Low cost)	[151]
